# Clinical Findings Documenting Cellular and Molecular Abnormalities of Glia in Depressive Disorders

**DOI:** 10.3389/fnmol.2018.00056

**Published:** 2018-02-27

**Authors:** Boldizsár Czéh, Szilvia A. Nagy

**Affiliations:** ^1^Neurobiology of Stress Research Group, Szentágothai Research Center, University of Pécs, Pécs, Hungary; ^2^Department of Laboratory Medicine, University of Pécs, Medical School, Pécs, Hungary; ^3^Department of Neurosurgery, University of Pécs, Medical School, Pécs, Hungary; ^4^MTA-PTE, Clinical Neuroscience MR Research Group, Pécs, Hungary; ^5^Pécs Diagnostic Centre, Pécs, Hungary

**Keywords:** astrocyte, depression, magnetic resonance imaging, microglia, oligodendrocyte, oligodendroglia, PET

## Abstract

Depressive disorders are complex, multifactorial mental disorders with unknown neurobiology. Numerous theories aim to explain the pathophysiology. According to the “gliocentric theory”, glial abnormalities are responsible for the development of the disease. The aim of this review article is to summarize the rapidly growing number of cellular and molecular evidences indicating disturbed glial functioning in depressive disorders. We focus here exclusively on the clinical studies and present the *in vivo* neuroimaging findings together with the postmortem molecular and histopathological data. Postmortem studies demonstrate glial cell loss while the *in vivo* imaging data reveal disturbed glial functioning and altered white matter microstructure. Molecular studies report on altered gene expression of glial specific genes. In sum, the clinical findings provide ample evidences on glial pathology and demonstrate that all major glial cell types are affected. However, we still lack convincing theories explaining how the glial abnormalities develop and how exactly contribute to the emotional and cognitive disturbances. Abnormal astrocytic functioning may lead to disturbed metabolism affecting ion homeostasis and glutamate clearance, which in turn, affect synaptic communication. Abnormal oligodendrocyte functioning may disrupt the connectivity of neuronal networks, while microglial activation indicates neuroinflammatory processes. These cellular changes may relate to each other or they may indicate different endophenotypes. A theory has been put forward that the stress-induced inflammation—mediated by microglial activation—triggers a cascade of events leading to damaged astrocytes and oligodendroglia and consequently to their dysfunctions. The clinical data support the “gliocentric” theory, but future research should clarify whether these glial changes are truly the cause or simply the consequences of this devastating disorder.

## Introduction

Depressive disorders are a leading cause of disease burden globally (Ferrari et al., 2013,^1^). There are effective therapeutic interventions available, but the currently existing antidepressant medications are far from being optimal as they have numerous side effects and large percentage of the patients do not respond to them (e.g., Anderson, 2000; Montgomery et al., 2002; Millan, 2006; Rush et al., 2006; Cipriani et al., 2009). There is an urgent need for new, faster acting, more effective drugs. Understanding the pathophysiology could help us in the development of novel therapies.

Despite extensive investigations, the exact neurobiological processes leading to depression are not fully understood. According to our present comprehension depressive disorders develop as a result of interactions between genetic predispositions and environmental factors (see e.g., Halldorsdottir and Binder, 2017; Bleys et al., 2018; Zhao et al., 2018; but also Culverhouse et al., 2018). The most widely accepted classic theory regarding the underlying neuropathology is the monoamine imbalance hypothesis, which emphasizes the role of disturbed monoamine neurotransmission in the synaptic cleft (Meyer et al., 2006; Belmaker and Agam, 2008; Nikolaus et al., 2009). However, it has become evident that this monoamine theory of depression does not explain the wide spectrum of macroscopic and microscopic structural changes that have been repeatedly documented in the brains of depressed patients. Currently, there are at least a dozen of theories aiming to explain the pathophysiology and here we focus on the “gliocentric hypothesis” of depression. The aim of this review is to summarize the clinical findings documenting glial changes in mood disorders based on the *in vivo* neuroimaging and the postmortem molecular and quantitative histopathological data.

Traditionally, the term “mood disorders” included both the bipolar and the depressive disorders, but now, in the DSM-5 there are separate sections for the Bipolar Disorders (BDs) and for the Depressive Disorders. All these terms mean larger categories and include several disorders. Here, we occasionally need to use the old terminology, since the majority of the studies have been done before the DSM-5. Most of the clinical studies discussed here involved patients either with Major Depressive Disorder (MDD) or with BD.

## Cellular Evidences for Glial Pathology

### Postmortem Quantitative Histopathological Data

We start with the postmortem histopathological findings since historically these were the first to reveal glial alterations in the brains of depressed individuals. The first reports were published in the late 90s. At that time, the scientific community was excited by the results of the *in vivo* imaging studies documenting reduced activity and volume shrinkage in the prefrontal cortex (PFC) of depressed patients (Drevets et al., 1997). To understand the cellular pathology behind these functional and morphological changes researchers started to carry out quantitative light microscopic analyses on postmortem PFC samples from depressed patients. The investigators expected to find changes in neuron numbers and morphology, but it turned out that in most cases the morphometric alterations affected glial cells. The first report found markedly reduced glial numbers in the ventromedial PFC (subgenual Brodmann’s area 24) in the brains of patients with MDD and BD (Öngür et al., 1998). The most prominent reduction of glial cell numbers was observed in subjects with familial MDD (24%) or BD (41%; Öngür et al., 1998). In their study, Öngür et al. (1998) used schizophrenic brains as psychiatric controls which had normal neuronal and glial cell numbers. These findings evoked great interest and were followed by numerous confirmatory reports. Reduced density of glial cells was found in the dorsolateral PFC (Cotter et al., 2002) and in the rostral and caudal orbitofrontal cortex of depressed patients (Rajkowska et al., 1999). A follow-up study by Rajkowska et al. (2001) revealed similarly reduced glial density in the dorsolateral PFC of patients with BD. More recent studies confirmed the findings on decreased density of glial cells in the anterior cingulate cortex (ACC; Brodmann area 24b; Gittins and Harrison, 2011). The histological studies on glial cell loss are supported by the recent molecular findings on altered microRNA (miRNA) levels in the brains of BD patients. For example Choi et al. (2017) extracted extracellular vesicles from the PFC (Brodmann area 24) and found increased levels of miR-149 exclusively in glial cells. Since miR-149 can inhibit glial cell proliferation thus, increased miR-149 expression might contribute to the glial cell number reduction.

The first studies focused on the different subareas of the PFC, but later the investigators analyzed other limbic areas and found similar glial changes. In the amygdala, reduced glial density and glia/neuron ratio was found in MDD (Bowley et al., 2002). This reduction was more pronounced in the left hemisphere and no change was found in neuron numbers (Bowley et al., 2002). In BD subjects, who did not receive mood stabilizer medication, significantly reduced glial cell numbers were found, in contrast to the cases who were treated with lithium or valproate (Bowley et al., 2002). This later finding indicated that medication may affect glial cell numbers (see also “Experimental Evidences That Psychotropic Medication Can Affect Glial Cell Numbers” section).

In the hippocampus, the first detailed human postmortem study found no evidence for neuronal abnormality, but the packing density of glial cells was significantly increased in all hippocampal subfields of patients with MDD (Stockmeier et al., 2004). A follow up study by the same group could not find any difference in the total cell numbers of neurons and glia in any hippocampal subarea (Cobb et al., 2013), but later they reported on reduced density of astrocytes in the hilus (Cobb et al., 2016).

Obviously, these postmortem histopathological studies have their limitations (the problem of tissue preservation and finding adequate controls), thus, some scientists question their scientific soundness. But in fact most of the *in vivo* neuroimaging and preclinical experimental data support the postmortem findings. Ideally, one should carry out longitudinal studies on well-characterized patients with repeated neuroimaging investigations and in the end one should do postmortem analysis on the brains of the same subjects (Stockmeier and Rajkowska, 2004). However, so far such studies are not available.

Finally, we should note here that there are also negative findings on glial cell numbers in the literature (e.g., Damadzic et al., 2001; Cotter et al., 2005; Khundakar et al., 2011; Rubinow et al., 2016).

#### Postmortem Evidences for Astrocytic Abnormalities

Most of the above mentioned histopathological studies used Nissl stained brain samples which is not suitable for the identification of the different glial subtypes. The later studies used immunohistochemistry which enabled us to specify which type of glial cells are affected by the disease. Numerous studies found evidences for altered number or disrupted integrity of astrocytes in depressed patients. For example, Miguel-Hidalgo et al. (2000) studied glial fibrillary acidic protein (GFAP)-immunoreactive astrocytes in the dorsolateral PFC of MDD subjects, and found a significant reduction of areal fraction and packing density of GFAP-positive cell bodies, but only in younger (30–45 years old) patients, whereas in older subjects (46–86 years old) these glial parameters tended to be greater compared to the corresponding controls. In line with these data, reduced GFAP protein levels were reported by a western blot study analyzing PFC samples from patients with MDD (Si et al., 2004). A more recent study investigated the coverage of blood vessels by aquaporin (AQ)-4-immunoreactive astrocytes in the PFC of MDD patients and found 50% reduction in the orbitofrontal gray matter (Rajkowska et al., 2013). In the same study, the coverage of blood vessels by GFAP-positive endfeet processes did not differ between the groups (Rajkowska et al., 2013). Others reported on reduced GFAP immunoautoradiography in the white matter of the ACC (Brodmann area 24b; Gittins and Harrison, 2011). Yet another study which did a very detailed analysis on the morphology of Golgi-impregnated astrocytes found that the fibrous astrocytes (in the white matter) had significantly larger cell bodies, as well as longer, more ramified processes in depressed suicide victims in the ACC (Brodmann area 24; Torres-Platas et al., 2011). Another study found decreased area fraction and increased cell clustering of GFAP expressing astrocytes in the postmortem white matter adjacent to the dorsolateral PFC (Brodmann area 9) of BD patients (Hercher et al., 2014).

Alterations of astrocytes have also been reported in the hippocampi of depressed patients. A study focusing on the density of GFAP-positive astrocytes found reduced density of astrocytes in the dentate hilus, but not in other hippocampal subareas (Cobb et al., 2016). Furthermore, this decrease was present only in those depressed patients who were not taking antidepressant medications, but not in subjects who were medicated (Cobb et al., 2016). Another group found reduced density of S100B-immunopositive astrocytes in the hippocampal CA1 pyramidal cell layer of MDD and BD patients (Gos et al., 2013). Reduced density of GFAP-immunoreactive astrocytes was also found in the amygdala of depressed patients (Altshuler et al., 2010) and downregulation of GFAP mRNA and protein expression was reported in the thalamus and caudate nucleus of depressed suicides (Torres-Platas et al., 2016).

Since it is increasingly acknowledged that astrocytes play a number of vital roles in the CNS (e.g., maintaining synaptic homeostasis, modulating glutamate metabolism, participating in signaling between neurons and glia, neurotrophic support, etc.) thus, a theory had been put forward proposing that depressive disorders are the consequence of the disturbed astrocytic functioning (see e.g., Wang et al., 2017). The summary of astrocytic abnormalities in depressed patients is shown on Table 1.

**Table 1:** Astrocytic abnormalities.

Type of disorder	*In vivo* imaging data	Postmortem molecular data	Postmortem cellular data
Major depressive disorder	PET studies document reduced glucose metabolism in the PFC (Baxter et al., 1989), amygdala (Abercrombie et al., 1998; Drevets et al., 2002b), thalamus (Su et al., 2014), and lateral temporal and parietal cortex (Drevets et al., 1992) Reduced *Glx* level detected by ^1^H-MRS (Yüksel and Öngür, 2010; Arnone et al., 2015) Increased lactate in the pregenual ACC (Ernst et al., 2017) Reduced metabotropic glutamate receptor 5 (mGluR5) binding in the PFC, cingulate cortex, insula, thalamus and hippocampus (Deschwanden et al., 2011)	Reduced GFAP protein expression in the PFC (Miguel-Hidalgo et al., 2000; Si et al., 2004), locus coeruleus (Chandley et al., 2013) and cerebellum (Fatemi et al., 2004) Reduced GFAP mRNA and protein expression in the thalamus and caudate nucleus (Torres-Platas et al., 2016) Reduced expression of astrocyte specific glutamate transporter genes (EAAT1, EAAT2) in the orbitofrontal cortex (Miguel-Hidalgo et al., 2010), dorsolateral PFC (Choudary et al., 2005; Zhao et al., 2016), hippocampus (Medina et al., 2013, 2016) and locus coeruleus (Bernard et al., 2011; Chandley et al., 2013) Downregulation of connexin 30 and 43 expressing genes in several brain areas (Nagy et al., 2017) Reduced gene expression of a gap junction protein (GJA1) in the hippocampus (Medina et al., 2016) Reduced gene expression of potassium (KCNJ10) and water channels (AQP4) in the hippocampus (Medina et al., 2016)	Larger cell bodies of astrocytes and longer, more ramified processes in the ACC (Torres-Platas et al., 2011) Reduced areal fraction and packing density of astrocytes in the DLPFC of young subjects (Miguel-Hidalgo et al., 2000) Increased areal fraction and packing density of astrocytes in the DLPFC of old subjects (Miguel-Hidalgo et al., 2000) Reduced coverage of blood vessels by AQ4-positive astrocytes in the orbitofrontal cortex (Rajkowska et al., 2013) Reduced GFAP-IR in the ACC white matter (Gittins and Harrison, 2011) Reduced density in the dentate hilus of non-medicated patients (Cobb et al., 2016) Reduced density of S100B-positive astrocytes in the hippocampal CA1 pyramidal cell layer (Gos et al., 2013) Reduced density in the amygdala (Altshuler et al., 2010) Reduced density of glutamine synthetase expressing astrocytes in specific cortical gray matter areas (Bernstein et al., 2015)
Bipolar disorder	Increased *Glx* and glutamate levels detected by ^1^H-MRS (Frye et al., 2007; Yüksel and Öngür, 2010; Gigante et al., 2012; Soeiro-de-Souza et al., 2015) Increased lactate in the cingulate gyrus (Dager et al., 2004)	Reduced expression of GFAP mRNA levels in the white matter of the ACC (Webster et al., 2005)	Increased clustering of astrocytes in the white matter adjacent to the DLPFC (Hercher et al., 2014) Reduced density of S100B-positive astrocytes in the hippocampal CA1 pyramidal cell layer (Gos et al., 2013)

*Abbreviations: ACC, anterior cingulate cortex; AQ, aquaporin; DLPFC, dorsolateral prefrontal cortex; EAAT, excitatory amino acid transporter; GFAP, glial fibrillary acidic protein; *Glx*, a composite measure of glutamate and glutamine in ^1^H-MRS studies; ^1^H-MRS, proton magnetic resonance spectroscopy; IR, immunoreactivity; PET, Positron-Emission Tomography; PFC, prefrontal cortex*.

#### Postmortem Evidences for Oligodendrocyte Abnormalities

Similarly to astrocytes, deficit of oligodendrocytes has been repeatedly documented in the PFC of MDD and BD patients. The first report was an electron microscopic study demonstrating ultrastructural evidences for apoptosis and necrosis of oligodendroglial cells in the prefrontal area 10 in BD (Uranova et al., 2001). In a follow-up study, the same group studied the numerical density of oligodendroglial cells in layer VI of the Brodmann area 9 in BD, MDD and healthy controls. They found a significantly reduced density of oligodendrocytes in BP and MDD patients (Uranova et al., 2004). Later, the same group demonstrated a prominent reduction in the number of perineuronal oligodendrocytes in layer III of the Brodmann area 9 in BP and MDD (Vostrikov et al., 2007). These findings are supported by more recent studies, using different cell counting techniques, but also reporting on reduced density of oligodendrocytes in the PFC Brodmann area 10 (Hayashi et al., 2011). Finally, a recent study found significantly decreased soma size of 2′,3′-Cyclic-nucleotide 3′-phosphodiesterase enzyme (CNPase)-immunoreactive oligodendrocytes in the ventral PFC white matter of MDD patients (Rajkowska et al., 2015). Complementing these quantitative findings, another study found reduced immunoreactivity of the myelin basic protein (an oligodendrocyte marker) in the anterior frontal cortex of depressed individuals who died by suicide (Honer et al., 1999). Another group found reduced density of S100B-immunopositive oligodendrocytes in the left hippocampal alveus of BD patients (Gos et al., 2013). More recently, a study found increased oligodendrocyte density in the postmortem white matter adjacent to the dorsolateral PFC (Brodmann area 9) of BD patients (Hercher et al., 2014). One should add here that there is a recent study which investigated the axonal myelin sheath in the genu of the corpus callosum and found that the myelin thickness was actually greater in MDD (Williams et al., 2015).

These neuroanatomical findings on oligodendroglial changes have been extended by a study which compared microarray metadata with the cytoarchitectural data. Correlation analysis between the genome-wide gene expression levels and cytoarchitectural traits revealed that 818 genes were significantly correlated with the decrease in the number of perineuronal oligodendrocytes in psychiatric subjects (Kim and Webster, 2010).

Based on these findings, it has been proposed that impaired oligodendrocyte functioning alters neuronal circuitry and by that leads to disrupted mood regulation in psychiatric disorders. For details of this concept see e.g., Edgar and Sibille (2012). The summary of oligodendrocyte abnormalities in depressed patients is shown on Table 2.

**Table 2:** Oligodendrocyte abnormalities.

Type of disorder	*In vivo* imaging data	Postmortem molecular data	Postmortem cellular data
Major depressive disorder	DTI studies report on robust reduction of fractional anisotropy in the corpus callosum and in several frontal and temporal regions (Alexopoulos et al., 2002; Taylor et al., 2004; Bae et al., 2006; Ma et al., 2007; Osoba et al., 2013; de Diego-Adeliño et al., 2014; Yamada et al., 2015; Jiang et al., 2017; Matsuoka et al., 2017) Altered MTR indicating reduced myelin integrity (Kumar et al., 2004; Gunning-Dixon et al., 2008; Chen et al., 2015; Jia et al., 2017) Reduced NAA/Cr ratio in the DLPFC white matter in first episode treatment-naive patients (Wang et al., 2012)	Reduced expression of 17 genes related to oligodendrocyte function in the temporal cortex (Aston et al., 2005) Altered expression of myelin-related mRNAs and proteins in the white matter of the ventral PFC (Rajkowska et al., 2015)	Reduced density in the PFC (Uranova et al., 2004; Hayashi et al., 2011) Reduced number in the PFC (Vostrikov et al., 2007) Reduced soma size in the white matter of the ventral PFC (Rajkowska et al., 2015) Reduced IR of myelin basic protein in the anterior frontal cortex (Honer et al., 1999) Greater axonal myelin thickness in the genu of the corpus callosum (Williams et al., 2015)
Bipolar disorder	DTI studies report on reduced fractional anisotropy in the cingulum, internal capsule, posterior corpus callosum, tapetum, and occipital white matter (Lu et al., 2011, 2012; Emsell et al., 2013; Sprooten et al., 2016; Ji et al., 2017) Greater mean diffusivity in the cingulum, corpus callosum, corona radiata, internal capsule, tapetum, and occipital white matter (Lu et al., 2011, 2012; Emsell et al., 2013; Sprooten et al., 2016; Ji et al., 2017) Altered MTR indicating reduced myelin integrity in the anterior cingulate and subgyral white matter (Bruno et al., 2004) Reduced NAA/Cr in the medial prefrontal white matter (Zhong et al., 2014)	Reduced expression of oligodendrocyte-related and myelination-associated genes (Tkachev et al., 2003) Increased CNPase protein levels in the white matter adjacent to the DLPFC (Hercher et al., 2014) Increased mRNA levels of the oligodendroglial markers (Olig1, Olig2) in the serum (Ferensztajn-Rochowiak et al., 2016)	Apoptosis and necrosis in the PFC (Uranova et al., 2001) Reduced density in the PFC (Uranova et al., 2004) Reduced number in the PFC (Vostrikov et al., 2007) Reduced density of S100B-positive oligodendrocytes in the left hippocampal alveus (Gos et al., 2013) Increased density in the white matter adjacent to the DLPFC (Hercher et al., 2014)

*Abbreviations: CNPase, 2′,3′-Cyclic-nucleotide 3′-phosphodiesterase enzyme; Cr, Creatine; DLPFC, dorsolateral prefrontal cortex; DTI, diffusion tensor imaging; IR, immunoreactivity; MTR, magnetization transfer ratio; NAA, N-acetylaspartate; PFC, prefrontal cortex*.

#### Postmortem Evidences for Activated Microglia

Microglial cells are the resident immune cells of the brain. Large body of evidence support the notion that neuroinflammation contributes to the pathophysiology of depression (Rosenblat et al., 2014) and numerous scientists proposed that microglial cells play a key role in the pathogenesis of depressive disorders (Brites and Fernandes, 2015; Yirmiya et al., 2015; Singhal and Baune, 2017). But in fact, so far only a handful of postmortem studies focused on microglial cells in the brains of depressed individuals. The first study to do that was a report on the presence of activated microglia in the hippocampal CA1 area using immunohistochemical staining with the HLA-DR antibody (Bayer et al., 1999). Later Steiner et al. (2008, 2011), provided further important evidences on microglial activation in various psychiatric illnesses. The summary of microglial changes in depressed patients is shown on Table 3.

**Table 3:** Microglial changes.

Type of disorder	*In vivo* imaging data	Postmortem molecular data	Postmortem cellular data
Major depressive disorder	Increased TSPO density in the PFC, ACC and insula detected by PET scan (Setiawan et al., 2015) TSPO availability was higher in the ACC and insula of patients with suicidal thoughts compared to patients without such intention (Holmes et al., 2018)	Up-regulated gene expression of Iba-1 and MCP-1 in suicides (Torres-Platas et al., 2014) Elevated cytokines (TNF-α, IL-1β, IL-6) and Toll-like receptors in the PFC of suicide victims (Pandey, 2017) Reduced expression of genes associated with microglia and glial cell functions in the DLPFC (Pantazatos et al., 2017)	HLA-DR-immunopositive activated microglia in the hippocampal CA1 area (Bayer et al., 1999) Significant microgliosis in the ACC, DLPFC and MD thalamus of suicide patients (Steiner et al., 2008) Significantly increased density of QUIN-positive cells in the sACC and aMCC (Steiner et al., 2008) Increased ratio of primed Iba-1-positive microglia in the white matter of the dorsal ACC in depressed suicides (Torres-Platas et al., 2014) Increased proportion of blood vessels surrounded by macrophages (Torres-Platas et al., 2014) Increased density of Iba-1-positive cells in contact with blood vessels in the dorsal PFC white matter of suicide victims (Schnieder et al., 2014)
Bipolar disorder	Elevated TSPO binding in the right hippocampus (Haarman et al., 2014)		

*Abbreviations: ACC, anterior cingulate cortex; aMCC, anterior midcingulate cortex; DLPFC, dorsolateral prefrontal cortex; HLA-DR, the major histocompatibility complex II protein; Iba-1, ionized calcium binding adaptor molecule 1; MCP-1, monocyte chemoattractant protein-1; MD, mediodorsal; PET, Positron-Emission Tomography; PFC, prefrontal cortex; QUIN, quinolinic acid; sACC, subgenual anterior cingulate cortex; TSPO, translocator protein-18 kDa*.

A more recent study analyzed Iba-1-immunoreactive microglial cells in the white matter of the dorsal ACC and found that the ratio of primed over ramified (“resting”) microglia was significantly increased in depressed suicides (Torres-Platas et al., 2014). In the same study, the proportion of blood vessels surrounded by macrophages was more than twice higher in depressed suicides than in controls (Torres-Platas et al., 2014). Consistent with these observations, gene expression analysis of Iba-1 and monocyte chemoattractant protein-1 (MCP-1; a chemokine involved in the recruitment of circulating monocytes), was significantly up-regulated in depressed suicides (Torres-Platas et al., 2014). Another study found greater density of Iba-1-immunoreactive cells in contact with blood vessels in the dorsal prefrontal white matter of suicide victims (Schnieder et al., 2014).

#### Experimental Evidences That Psychotropic Medication Can Affect Glial Cell Numbers

Ideally, a postmortem study investigating cellular changes in the brains of depressed individuals should use tissue samples from medication free patients. However, nowadays it is very difficult to get such samples, because most patients with depressive disorder or BD receive long-term treatment with various drugs. Thus, when a human postmortem study reports on altered cell numbers in specific brain areas, the next question to be answered: is this change due to the illness, or is this the result of the medication? To answer this question, several groups examined neuronal and glial cell numbers in the brains of experimental animals subjected to antidepressant, antipsychotic or mood stabilizer treatment. For example, a study treated macaque monkeys with antipsychotic drugs such as haloperidol and olanzapine (at doses producing plasma levels in the therapeutic range) and found that glial cell numbers were significantly reduced in the parietal cortex (Konopaske et al., 2007). In a follow up study, the same group reported that these glial changes affected mainly the astrocytes, since a significant loss of astrocytes were found in addition to a non-significant reduction in oligodendrocyte cell number in the antipsychotic-treated monkeys (Konopaske et al., 2008). Haloperidol and olanzapine had equivalent glia-reducing effect (Konopaske et al., 2008). A more recent study investigated the effect of lithium treatment on glial cell numbers in the PFC and dentate gyrus of mice (Rajkowska et al., 2016). Lithium is a widely used mood stabilizer and many patients with BD receive long-term lithium treatment. This experimental study found that lithium treatment can increase the total numbers of neurons and glia in the dentate gyrus and also the density of astrocytes in the PFC of mice (Rajkowska et al., 2016). Besides lithium, valproic acid is a classic choice to treat manic or mixed episodes of BD. There is increasing evidence that chronic valproic acid treatment can influence myelination and network connectivity (Rosenzweig et al., 2012). We also did an experimental study to examine the effect of an antidepressant drug (fluoxetine, a selective serotonin reuptake inhibitor) on astrocytes in an animal model for depression. We found that fluoxetine could reverse the chronic stress-induced loss of astrocytes in the hippocampus of the animals (Czéh et al., 2006). Furthermore, treatment with psychoactive agents not only affects glial cell numbers, but also influences cellular morphology. It has been shown that fluoxetine treatment can increase the plasticity of astrocytic end-feet processes and enhance their numbers (Di Benedetto et al., 2016). Similarly to that, a study using a non-human primate model of self-injurious behavior reported that naltrexone treatment can reverse astrocyte atrophy associated with self-harm (Lee et al., 2015).

Adult hippocampal neurogenesis in the dentate gyrus is a cellular process that has been linked to the pathophysiology of depressive disorders (see e.g., Miller and Hen, 2015). Numerous studies document that antidepressant treatments can stimulate adult hippocampal neurogenesis and parallel to this several studies investigated the effect of antidepressant treatment on gliogenesis in the CNS of experimental animals. Several reports document increased gliogenesis in the PFC of adult rats after antidepressant or antipsychotic treatment (Kodama et al., 2004; Czéh et al., 2007). Likewise, electroconvulsive treatment stimulates gliogenesis in the PFC (Madsen et al., 2005; Öngür et al., 2007), hippocampus (Wennström et al., 2006), and amygdala (Wennström et al., 2004). These treatments stimulated the proliferation of NG2-positive glial cells. In contrast to the stimulating effect of antidepressants, lithium treatment appears to inhibit the proliferation of NG2 cells in the hippocampal dentate hilus, amygdala and corpus callosum (Orre et al., 2009).

In summary, experimental data indicate that drugs, characteristically used to treat MDD and BD, can all affect glial cell numbers, cellular morphology, gliogenesis and probably also myelination. This should be kept in mind when we interpret the postmortem clinical findings, because most brain samples originate from individuals who received long-term medications during the course of their illness.

### *In Vivo* Imaging Studies

*In vivo* neuroimaging is a valuable tool to examine glial alterations in depressive disorders. The volumetric magnetic resonance imaging (MRI) studies reveal reduced volumes of specific brain areas in depression (e.g., van Tol et al., 2010; Grieve et al., 2013). These volume changes are likely to be the gross consequence of the glial and/or neuronal cell loss. There are numerous novel *in vivo* imaging methods which can be employed to examine brain structure and function. These methods include the diffusion tensor imaging (DTI), functional magnetic resonance imaging (fMRI), proton magnetic resonance spectroscopy (^1^H-MRS) and positron emission tomography (PET) imaging. Here, we summarize the *in vivo* clinical findings documenting glial abnormalities in patients with depressive disorders.

#### Astrocytic Abnormalities

Astrocytes carry out a large variety of important cellular functions for the neuronal microenvironment such as the regulation of glucose metabolism, neurotransmitter uptake (particularly glutamate, the major excitatory neurotransmitter), regulation of synaptic development and maturation and maintenance of the blood brain barrier (Kettenmann and Ransom, 2005). In PET imaging, the uptake of the radioactive ^18^F-deoxyglucose by astrocytes is associated with neuronal activity (Magistretti and Pellerin, 1999). A well replicated finding in PET studies is the reduced glucose metabolism in the PFC of MDD patients (Baxter et al., 1989). Typically this is more pronounced in depressed periods and slightly normalized in the remission phase (Martinot et al., 1990; Drevets et al., 2002a). Mounting evidence supports the existence of abnormal glucose metabolism in other brain regions including the amygdala (Abercrombie et al., 1998; Drevets et al., 2002b), thalamus (Su et al., 2014), and the lateral temporal and parietal cortex (Drevets et al., 1992), however these later ones are less consistent.

Numerous ^1^H-MRS studies examined glutamate, glutamine and *Glx* (a composite measure of glutamate and glutamine) levels in the context of mood disorders (reviewed by Yüksel and Öngür, 2010). These studies find a highly consistent pattern of *Glx* level reductions in MDD and elevations in BP (Yüksel and Öngür, 2010). Furthermore, the available data suggest that in depressive states reduced glutamine/glutamate ratio can be detected, whereas in mania an elevated glutamine/glutamate ratio is present (Yüksel and Öngür, 2010; Luykx et al., 2012; Arnone et al., 2015). These metabolic changes are likely to be the consequences of the altered astrocytic functioning. A recent meta-analysis based on 17 studies using ^1^H-MRS revealed an exclusive reduction in *Glx* levels in the PFC of MDD patients (Arnone et al., 2015). The same meta-analysis reported that there was no change in glutamate levels and other metabolite levels were also not altered (Arnone et al., 2015). Other investigation found normal or slightly elevated glutamate and glutamine levels in remitted MDD (Bhagwagar et al., 2007), which suggests that *Glx* may be restored or compensated after successful treatment (Michael et al., 2003; Pfleiderer et al., 2003).

Several PET studies investigated tracer binding to the metabotropic glutamate receptor 5 (mGluR5) which is a key component of the glutamatergic system and expressed by neurons and astrocytes (Biber et al., 1999). A study by Deschwanden et al. (2011) used the radiotracer [^11^C]ABP688 and found reduced mGluR5 binding in the PFC, cingulate cortex, insula, thalamus, and hippocampus of MDD patients. However, more recently a study using a different radiotracer, [^18^F]FPEB, could not confirm this finding (Abdallah et al., 2017).

In case of BD patients, the picture is less ambiguous, because numerous studies consistently report on increased *Glx* and glutamate levels in several brain areas (Frye et al., 2007; Gigante et al., 2012; Soeiro-de-Souza et al., 2015). A study which involved both melancholic and non-melancholic subtypes of BD found that *Glx* and glutamate concentrations were significantly higher in the non-melancholic subtype (Frye et al., 2007). Furthermore, patients who responded to lamotrigine treatment had reduced glutamine concentration (Frye et al., 2007). Notably, up to 80% of glutamate is compartmentalized in neurons while glutamine is synthetized mainly in astrocytes (Maddock and Buonocore, 2012; Ramadan et al., 2013), hence, both astrocytic and neuronal dysfunction can lead to changes in glutamine and glutamate levels (see also “Molecular Evidences From Clinical Studies: Astrocytic Abnormalities” section).

Lactate is an important metabolite in the brain with unknown function. Astrocytes contribute to lactate metabolism in the brain (Mächler et al., 2016; Mason, 2017). Furthermore, lactate can be processed by mitochondrial oxidative metabolism (Pellerin et al., 2007) and recent theories suggest mitochondrial dysfunction in depressive disorders (Manji et al., 2012; Klinedinst and Regenold, 2015). Supporting this concept, elevated gray matter lactate was found in the cingulate gyrus of BD patients (Dager et al., 2004) and in the pregenual ACC of patients with MDD (Ernst et al., 2017).

It is well known, that astrocytic glycolysis and oxidative phosphorylation results in a clear signal change in fMRI measurements (Rossi, 2006; Attwell et al., 2010). Numerous studies document fMRI alterations in fronto-cortical areas of MDD patients (e.g., Grimm et al., 2008; Mulders et al., 2015; Davey et al., 2017), but it is not known which cell type is responsible for the alterations. In the PFC, the coverage of blood vessels by astrocytic endfeets is markedly reduced in patients with MDD suggesting that the blood flow and glucose uptake by astrocytes might be impaired (Rajkowska et al., 2013). This neuroanatomical alteration could consequently result in fMRI signal change. Plenty of evidences document that antidepressant treatment (e.g., fluoxetine, paroxetine) positively affect astrocytic function by regulating glucose metabolism (Kennedy et al., 2001; Allaman et al., 2011; Czéh and Di Benedetto, 2013) and consequently cause fMRI signal change (Harris and Reynell, 2017). Interestingly, antidepressant treatment can affect the BOLD response to positive and negative emotional stimuli differently especially in frontal brain areas (Ma, 2015).

#### Oligodendrocyte Abnormalities

Oligodendroglia provides support and insulation to axons by creating the myelin sheath. Consequently, oligodendrocyte pathology can cause abnormal development, demyelination or reduction of myelinated axons. Oligodendrocyte abnormalities can be investigated *in vivo* using DTI. DTI was specifically developed to investigate the integrity of white matter, where axons and myelin sheaths form longitudinal axes leading to greater water diffusion along the tracts and restricted molecular displacement perpendicularly. Fractional anisotropy (FA) measures the magnitude of directionally varying diffusion restriction effect, while mean diffusivity (MD) represents the average diffusion coefficient in all direction.

A significant number of DTI studies found robust FA reductions in the corpus callosum and in several frontal and temporal regions (e.g., Alexopoulos et al., 2002; Taylor et al., 2004; Bae et al., 2006; Ma et al., 2007; de Diego-Adeliño et al., 2014; Yamada et al., 2015; Jiang et al., 2017) indicating white matter microstructural abnormalities in depressed patients. Later studies found similar microstructural abnormalities in the anterior callosal fibers connecting bilateral frontal cortices in patients with MDD (Yamada et al., 2015; Matsuoka et al., 2017). Another study reported on significant FA deficits only in the right parietal white matter in depressed patients as well as white matter changes of specific thalamic tracts (Osoba et al., 2013).

DTI studies document abnormal white matter microstructure in BD patients as well. Untreated bipolar patients with first episode psychosis showed lower FA in several white matter tracts such as the cingulum, internal capsule, posterior corpus callosum, tapetum and occipital white matter (Lu et al., 2011). At the same time, BD patients have greater MD in the cingulum, corpus callosum, corona radiata, internal capsule, tapetum and occipital white matter including posterior thalamic radiation, inferior longitudinal fasciculus/inferior fronto-occipital fasciculus (Lu et al., 2011). Numerous follow up studies confirmed these data (Lu et al., 2012; Emsell et al., 2013; Sprooten et al., 2016; Ji et al., 2017). Similar, but more subtle white matter microstructural changes were detected in unaffected siblings as well (Sprooten et al., 2013, 2016). A recent study reported that these alterations are less pronounced in euthymic BD patients and that lithium treatment can counteract the white matter microstructural abnormalities (Haarman et al., 2016b).

There are other imaging methods to quantitatively evaluate myelin related white matter alterations. Magnetization transfer imaging is based on the interaction of unbound protons and protons bound to macromolecules. Myelin bound protons associated with protein and lipid macromolecules are not measurable by conventional MRI. During magnetization transfer imaging an off-resonance pulse is applied to partially saturate the macromolecular pool and produce contrast between tissues. This suppresses the water signal which can be measured by magnetization transfer ratio (MTR) and associated with macromolecular concentration. Hence, increased MTR in the white matter indicates remyelination, whereas decreased MTR indicates demyelination (Chen et al., 2008). Besides the gray matter MTR changes (Chen et al., 2015; Jia et al., 2017), the white matter also depicts alterations in depressive disorders. Reduced myelin integrity was suggested in late-life depression where decreased MTR was found in multiple left hemisphere frontostriatal, limbic areas, occipital white matter and in the genu and splenium of the corpus callosum (Kumar et al., 2004; Gunning-Dixon et al., 2008). Subtle abnormalities were presented in the anterior cingulate and subgyral white matter in patients with BD reflecting myelin changes and/or reduced axon density (Bruno et al., 2004).

Growing number of evidences indicate that cerebral white matter lesions contribute to the pathophysiology of depression. Recent studies demonstrate that the volume of periventricular and deep white matter lesions are differently associated with depressive symptoms (Tully et al., 2017). These are also present in suicidal behavior of MDD and BD patients (Pompili et al., 2008; Grangeon et al., 2010). Despite their similar appearance, some authors suggest that lesions in different brain areas may have distinct origin and functional consequences. Accordingly, periventricular white matter lesions have been found to have lower MTR than the ones in deep white matter, suggesting different underlying pathology and mechanisms (Spilt et al., 2006). It is argued that these white matter lesions should result in impaired connectivity of different brain regions. Lower white matter microstructural integrity (measured by DTI) and altered brain function (measured by fMRI) were found in white matter lesions (Smagula and Aizenstein, 2016). Others reported more widespread changes in white matter connectivity and lesions in BD compared to MDD (Cardoso de Almeida and Phillips, 2013). Overall, these data suggest that white matter lesions contribute to the development of depressive disorders.

^1^H-MRS is another *in vivo* method to assess cellular integrity and functioning in the brains of psychiatric patients (see e.g., Yildiz-Yesiloglu and Ankerst, 2006; Caverzasi et al., 2012). With this method one can measure the concentration of various brain metabolites, typically, *N*-acetylaspartate (NAA), Choline (Cho), *myo*-inositol (mI), Glutamate (Glu)/Glutamine (Gln) and Creatine (Cr). *NAA* plays a role in various cellular functions such as osmoregulation, energy homeostasis and possibly also in myelin production (Moffett et al., 2007). NAA levels in the CNS provide information on the functioning of both neurons and oligodendrocytes. For example, significantly lower NAA to Cr ratio was found in the dorsolateral prefrontal white matter in first episode treatment-naive patients with MDD (Wang et al., 2012). Similarly, reduced NAA/Cr ratio was found in patients with BD in the medial prefrontal white matter (Zhong et al., 2014). These results indicate that, *in vivo* NAA changes do not reflect neuronal alterations alone, but also glial dysfunctions, both in MDD and BD.

PET is a well-established method to detect ongoing demyelination and remyelination processes *in vivo* (Stankoff et al., 2006, 2011; Wang et al., 2009; Wu et al., 2010; Tiwari et al., 2016), but to our best of knowledge so far no one investigated myelin related alterations in depressive disorders using PET imaging.

#### Microglial Abnormalities

Neuroinflammatory processes have been repeatedly implicated in the pathophysiology of depression. As noted earlier, microglia is a key component of the immune system and seems to contribute to the pathophysiology of depression (Eyre and Baune, 2012). Activated microglia and neuroinflammation can be studied with PET imaging, using dedicated radiopharmaceuticals targeting the translocator protein-18 kDa (TSPO). In MDD, TSPO-specific V_T_ (an index of TSPO density) was significantly increased by the magnitude of 30% in the PFC, ACC and insula using [^18^F] FEPPA radiopharmaceutical (Setiawan et al., 2015). In euthymic BD, elevated TSPO binding was found by the [^11^C] PK-11195 ligand in the right hippocampus compared to healthy controls (Haarman et al., 2014). However, another study using [^11^C] PBR28 found no significant difference between mild-to-moderate MDD patients and controls across a range of gray matter regions (Hannestad et al., 2013). Interestingly, Holmes et al. (2018) found that TSPO availability (measured by [^11^C] PK-11195 radioligand) was higher in the ACC and insula of MDD patients with suicidal thoughts compared to patients without such intention. They also provided the first evidence that increased TSPO may be more associated with suicidality than the MDD diagnosis itself (Holmes et al., 2018). Although the applied methodology and the binding affinity patterns of TSPO PET ligands can be different across the studies, these findings are the most compeling evidences for ongoing neuroinflammation, and for microglial activation during depressive episodes.

It has been suggested that ^1^H-MRS can also detect activated microglia-induced metabolic changes associated with neuroinflammation in depressive states (Haroon and Miller, 2017). Inflammatory cytokines have been shown to influence glutamate metabolism in MDD, but in the literature one can find contradictory findings. The majority of studies have shown decreased *Glx* in the ACC, amygdala, hippocampus and in different subregions of the PFC (Yüksel and Öngür, 2010), while others reported increased glutamate in other brain areas (Sanacora et al., 2004). After various treatment protocols, including a serotonin reuptake inhibitor drug (citalopram, Taylor et al., 2008), electroconvulsive therapy (Zhang et al., 2013) and sleep deprivation (Murck et al., 2009) the concentration of *Glx* can normalize in the brains of MDD patients.

Studies involving BD patients have consistently reported increased *Glx* levels both in medication free and treated patients (Gigante et al., 2012). Furthermore, a recent study reported that neuronal integrity markers NAA and *N*-acetyl-aspartyl-glutamate correlated with microglia activation (measured by TSPO). Based on this, it was proposed that some microglia can induce apoptosis while others stimulate adult neurogenesis (Haarman et al., 2016a). Finally, we should emphasize that methodological heterogeneity of the ^1^H-MRS studies (e.g., absolute concentration or ratio measurement, sample size, field strength, voxel location and geometry, etc.) may account for the contradictory results (Yüksel and Öngür, 2010).

Several studies reported that therapeutic administration of IFN-alpha (e.g., to treat viral infections) can induce profound inflammatory response and interacts with serotonin metabolism and increases glutamate which all in turn can result in depressive mood (Raison et al., 2009; Haroon et al., 2014). INF-alpha administration was associated with increased activity in the dorsal ACC and linked to impaired cognitive performance (Capuron et al., 2005). A recent resting-state fMRI study demonstrated that increased plasma concentration of C-reactive protein (a marker for inflammation) was associated with decreased connectivity between the ventral striatum and ventromedial PFC, which in turn correlated with increased anhedonia (Felger et al., 2016). Another resting-state fMRI study which used large multisite sample showed that depression can be subdivided into four subgroups by dysfunctional connectivity patterns in limbic and frontostriatal networks and also predicted the responsiveness to transcranial magnetic stimulation therapy (Drysdale et al., 2017).

## Molecular Evidences from Clinical Studies

### Astrocytic Abnormalities

There are numerous direct and indirect molecular evidences indicating altered astrocyte functioning in depressive disorders. Several studies reported an age-dependent reduction in the expression level of GFAP in the PFC of MDD patients (Miguel-Hidalgo et al., 2000; Si et al., 2004). Reduced expression of GFAP mRNA levels were found in the white matter of the ACC in bipolar patients (Webster et al., 2005). Reduced GFAP expression was found also in the locus coeruleus (Chandley et al., 2013) and in the cerebellum (Fatemi et al., 2004) of patients with MDD.

GFAP corresponds to GFAP—an intermediate filament protein—which has become the prototypical marker for immunohistochemical identification of astrocytes. The exact functional role of GFAP is unknown, but it has been suggested to play a role in astrocyte-neuron interactions and glial scar formation upon CNS injury (Sofroniew and Vinters, 2010) as well as in intracellular vesicle trafficking (Potokar et al., 2010).

Astrocytes are key cellular elements regulating glutamate concentrations both in the synaptic cleft and in the extracellular space (Haydon and Carmignoto, 2006). Numerous MRS studies reported on altered levels of glutamate-related metabolites in mood disorders (see “*In Vivo* Imaging Studies: Astrocytic Abnormalities” section). In line with this, a postmortem study reported reduced densities of glutamine synthetase expressing astrocytes in specific cortical gray matter areas in MDD (Bernstein et al., 2015). Glutamine synthetase catalyzes the ATP-dependent condensation of ammonia and glutamate to form glutamine, and by that plays a central role in glutamate and glutamine homoeostasis. In addition to this, several studies documented reduced expression of astrocyte specific glutamate transporter genes (EAAT1, EAAT2 or SLC1A2, SLC1A3) in the orbitofrontal cortex (Miguel-Hidalgo et al., 2010), dorsolateral PFC (Choudary et al., 2005; Zhao et al., 2016), hippocampus (Medina et al., 2013, 2016) and locus coeruleus (Bernard et al., 2011; Chandley et al., 2013) of patients with MDD. In sum, there are ample evidences from different sources that glutamate and glutamine metabolism is impaired in depressed patients as well as in manic episodes (Öngür et al., 2008), which suggests aberrant functioning of astroglial cells in depressed patients (Haroon et al., 2017). We should also cite here a recent preclinical study which demonstrated that blockade of the astrocytic glutamate transporter (GLT-1) in the central amygdala can induce anhedonia and anxiety in rats (John et al., 2015).

Other astrocytic functions are also disturbed in depressive disorders. For example altered expression of glial gap junction proteins have been reported, indicating changes in astrocyte-astrocyte communication. Downregulation of connexin 30 and 43 expressing genes were found in several brain areas (Nagy et al., 2017), and reduced gene expression of the gap junction protein (GJA1) was reported in the hippocampus (Medina et al., 2016).

Finally, there are evidences on reduced gene expression of potassium and water channels (KCNJ10, AQP4) in the hippocampus of depressed patients (Medina et al., 2016).

### Oligodendrocyte Abnormalities

The first study to document changes in oligodendrocyte-specific genes in the brains of psychiatric patients was done by Tkachev et al. (2003). They used differential display PCR, quantitative PCR and microarray analysis which provided evidences on reduced oligodendrocyte-related and myelination-associated gene expression in the brains of patients who had schizophrenia and BD. Later, Aston et al. (2005) reported on altered gene expression in the temporal cortex of MDD patients, which suggested oligodendroglial abnormalities. They used Affymetrix HgU95A microarray analysis and found a significant decrease in the expression of 17 genes related to oligodendrocyte function. Eight of these genes encode structural components of myelin (CNP, MAG, MAL, myelin oligodendrocyte glycoprotein (MOG), MOBP, PMP22, PLLP, proteolipid protein 1 (PLP1)), five other genes encode enzymes involved in the synthesis of myelin constituents (ASPA, UGT8), or regulate myelin formation (ENPP2, EDG2, TF, KLK6). SOX10, which encodes a transcription factor regulating other myelination-related genes, was also down regulated. OLIG2, a transcription factor specific for oligodendrocytes and oligodendrocyte precursors and ERBB3, which is involved in oligodendrocyte differentiation were also down regulated together with a number of genes involved in axonal growth and synaptic function (Aston et al., 2005). Overall these gene expression changes suggest disturbed neuronal communication and signal transduction mechanisms.

A recent postmortem study examined myelin-related mRNA and protein expression in the white matter of the ventral PFC in MDD patients (Rajkowska et al., 2015). Quantitative RT-PCR revealed significantly reduced expression of PLP1 mRNA and increased expression of mRNA for CNPase, MOG and oligodendrocyte transcription factors 1 (Rajkowska et al., 2015). The expression of CNPase protein was also significantly decreased in MDD. These data suggests molecular mechanisms for the degeneration of cortical axons and dysfunctional maturation of oligodendrocytes in MDD (Rajkowska et al., 2015). In BD however, increased CNPase protein levels were reported in the white matter adjacent to the dorsolateral PFC (Brodmann area 9; Hercher et al., 2014).

Interestingly, increased mRNA levels of the oligodendroglial markers, Olig1 and Olig2, have been found also in the serum of BD patients (Ferensztajn-Rochowiak et al., 2016).

### Microglial Abnormalities

Numerous studies report on elevated inflammatory markers in a subgroup of patients with MDD and BD (for reviews see e.g., Rao et al., 2010; Rosenblat et al., 2014; Réus et al., 2015; Miller and Raison, 2016; Sayana et al., 2017). However, most of the studies report on cytokine abnormalities in the periphery (in the serum) of depressed patients. Direct evidences on elevated cytokines and Toll-like receptors in postmortem brain tissue of suicide victims have been reported recently by Pandey (2017). This study found significantly increased mRNA and protein expression of TNF-α, IL-1β, IL-6 and Toll-like receptors in the PFC of suicide victims (Pandey, 2017). Pantazatos et al. (2017) used next generation sequencing for whole-transcriptome profiling (RNA-seq) to identify genes, miRNA species, and molecular pathways that are altered in the dorsolateral PFC of MDD patients. They found altered immune-related gene expression in depression and suicide, and a markedly lower expression of genes associated with microglia and glial cell functions (Pantazatos et al., 2017).

## Summary and Future Directions

There is ample evidence that glial abnormalities are present in the brains of depressed individuals. These glial changes affect all major glial cell types, astrocytes, microglia and oligodendrocytes and are detectable at multiple levels: at molecular, cellular and network level. Notably, we could not find any clinical studies on NG2-psoitive glia in the context of depressive disorders.

Here, we gathered the clinical observations and we did not consider the potential functional consequences, mainly because a number of recent excellent reviews discuss these issues. Astrocytes carry out a large number of vital cellular functions (e.g., Sofroniew and Vinters, 2010), their functional deficits can lead to various malfunctions, most prominently to disturbed glutamate and ion homeostasis, and to synaptic dysfunctions (see e.g., Rajkowska and Stockmeier, 2013; Jun et al., 2014; Verkhratsky and Parpura, 2016; Haroon et al., 2017; Sild et al., 2017; Wang et al., 2017). The main function of oligodendrocytes is to provide support and insulation to axons, thus, their dysfunction can lead to disrupted neuronal network connectivity and communication and consequently result in psychopathology (Menon, 2011; Edgar and Sibille, 2012). Plenty of evidences suggest neuroimmune etiology (Yirmiya et al., 2015; Miller and Raison, 2016; Haroon et al., 2017) or at least disturbed immune response regulation in a subgroup of depressed individuals (Mechawar and Savitz, 2016) and activated microglia is one player in this complex multifaceted process.

There are two key questions that should to be clarified in the future: (1) Are these glial changes represent the cause or the consequence of the disease? (2) Do these glial abnormalities relate to each other, or are they present in different subgroup of patients? Some argue that the different glial abnormalities are connected to each other. It has been proposed that the microglia mediated inflammatory processes can damage the oligodendrocytes and disrupt glutamate homeostasis by impairing astrocytic functions (Mechawar and Savitz, 2016; Haroon et al., 2017). Prospective clinical studies collecting blood samples for biomarker analysis and combining *in vivo* neuroimaging data with postmortem histopathological analysis could help to answer these important questions. The clinical studies should also investigate putative changes affecting NG2-expressing glial cells. Preclinical studies, targeting specific glial cell types in transgenic animals (Birey et al., 2015) can also provide valuable insights.

## Author Contributions

BC had the concept and wrote the parts on the cellular and molecular findings. SAN wrote the parts on the *in vivo* imaging data.

## Conflict of Interest Statement

The authors declare that the research was conducted in the absence of any commercial or financial relationships that could be construed as a potential conflict of interest.

## Footnotes

^1^http://www.who.int/mediacentre/factsheets/fs369/en/
